# Genome-wide identification and characterization of the lettuce GASA family in response to abiotic stresses

**DOI:** 10.1186/s12870-023-04101-5

**Published:** 2023-02-22

**Authors:** Sun Ho Lee, Jin Seok Yoon, Woo Joo Jung, Dae Yeon Kim, Yong Weon Seo

**Affiliations:** 1 Department of Plant Biotechnology, Korea University, Seoul, 02841 Republic of Korea; 2 Ojeong Plant Breeding Research Center, Korea University, Seoul, 02841 Republic of Korea; 3 Institute of Life Science and Natural Resources, Korea University, Seoul, 02841 Republic of Korea; 4 Department of Plant Resources, College of Industrial Science, Kongju National University, Yesan, 32439 South Korea

**Keywords:** Heat stress, *Lactuca sativa* L, Shoot apical meristem, *cis*-element, Expression analysis

## Abstract

**Background:**

Lettuce is one of the most extensively farmed vegetables in the world, and it prefers cool growing conditions. High temperatures promote premature bolt formation, reducing quality and yield. The gibberellic acid-stimulated *Arabidopsis* (*GASA*) family genes play critical roles in plant growth, development, and stress responses. However, the biological functions of GASA proteins in lettuce have yet to be thoroughly investigated.

**Results:**

Using genome-wide analysis, 20 *GASA*s were identified in lettuce including, three groups of LsGASA proteins based on the phylogenetic analysis. Except for one, all GASA proteins included a conserved GASA domain with 12 cysteine residues. *Cis*-element analysis showed that *LsGASA*s were closely associated with light, phytohormones, and stress resistance. Five segmental and three tandem duplication events were observed in the *LsGASA* family based on duplication analysis. *GASA* synteny analysis among lettuce, *Arabidopsis,* tobacco, and rice revealed that *LsGASA5* is highly collinear with all species. Six of the 20 *LsGASA* showed increased expression patterns at specific time points in the shoot apical meristem when subjected to heat stress. According to gene expression analysis, the majority of *GASA* were highly expressed in flowers compared to other organs, and six *GASA* exhibited highly increased expression levels in response to NaCl, abscisic acid, and gibberellin treatment. Furthermore, LsGASA proteins are predominantly found in the plasma membrane and/or the cytosol.

**Conclusions:**

This study provides a comprehensive characterization of *LsGASA* genes for their diversity and biological functions. Moreover, our results will be useful for further studies on the function of lettuce *GASA* in abiotic stress- and heat-induced bolting signaling.

**Supplementary Information:**

The online version contains supplementary material available at 10.1186/s12870-023-04101-5.

## Introduction

The *GASA/GAST* gene family is found in a variety of plant species and includes a signal peptide at the N-terminus and a conserved domain with 12 cysteine residues known as the GASA domain (PF02704) at the C-terminus, [1]. *GAST1* (GA-stimulated transcript 1) was first characterized in tomato (*Solanum lycopersicum* L.) [2]. Other *GASAs* have been identified in several species, including 19 in tomato (*Solanum lycopersicum* L.) [3], 15 in *Arabidopsis* (*Arabidopsis thaliana* L.) [1], 9 in rice (*Oryza sativa* L.) [4], 17 in cacao (*Theobroma cacao* L.) [5], 37 in wheat (*Triticum aestivum* L.) [6], 14 in grapevine (*Vitis vinifera* L.) [7], and 18 in tobacco (*Nicotiana tabacum* L.) [8].

GASA proteins are essential for many biological processes and play a key role in plant development, such as stem elongation [9], flowering [10], root development [11], fruit ripening [12], and seed development [7, 13]. Several studies have reported that *GASA* is involved in phytohormone responses, including gibberellin (GA), abscisic acid (ABA), auxins (IAA), brassinosteroids (BR), and salicylic acid (SA) [14, 15]. For instance, *GASA4* and *GASA6* are downregulated by JA, ABA, and SA in *Arabidopsis* but abundantly expressed by GA, brassinosteroids, auxins, and cytokinins [15]. *GASA1* expression analysis in wheat revealed that ACC, ABA, and MeJA were responsible for its induction [16]. Several interactions between *GASA* and DELLAs, which are negative regulators of GA signaling, have been discovered which imply that *GASA* significantly contributes to GA signaling [17]. *GsGASA1* is involved in suppressing root development through the accumulation of DELLA proteins under cold stress [18]. Other studies have reported that *GASA* influences resistance to abiotic stress [19, 20]. For instance, it has been demonstrated that *Arabidopsis* specimens that overexpress FsGASA4 (*Fagus sylvatica*) and *TaGASR1* are more resistant to oxidation, and salt during seed germination and seedling growth [21] and heat stress resistance, respectively [16].

Lettuce is a cool season crop with an optimal growth temperature range of 15–25 °C that is mostly used in salads. In recent years, the consumption of lettuce has increased significantly owing to its health benefits, such as allelopathic activity and a variety of bioactive phytochemical nutrients including anthocyanins, phenolic acids, and carotenoids [22, 23]. Harsh environmental conditions typically decrease lettuce production [24].

Environmental factors such as heat stress restrict plant growth and productivity [25]. The transition from the vegetative to the reproductive stage is accelerated under heat stress, which directly results in bolting and flowering [26]. *FLOWERING LOCUS T (FT)* and *SUPPRESSOR OF OVEREXPRESSION OF CONSTANS1 (SOC1),* which play important roles in the flowering molecular pathway, were upregulated by heat treatment and delayed bolting and flowering were observed after the knockdown of *soc1* and *ft* by RNAi from lettuce [27, 28]. Bolting lettuce results in a decreased leaf nutrient content, texture, and productivity. Therefore, studies related to delayed bolting and flowering are required to maintain suitable yield and quality.

Elucidation of the molecular regulatory network of heat-induced bolting in lettuce might be crucial for the development of heat-resistant lettuce since lettuce bolting time affects yield, quality, and consumer preference. Although *GASA*s are important regulators of plant development, heat-induced bolting with *GASA* expression has not yet been studied in lettuce. In this study, we identified a novel *GASA* family in the lettuce genome and examined their gene structure, conserved protein motifs, *cis*-acting elements, chromosomal localization, and synteny. The expression of *LsGASA* in the apical meristem tissues in response to heat stress was investigated. In addition, transcript levels of heat induced *GASA* were analyzed in response to abiotic stress and hormones, and subcellular localization was observed.

## Results

### Identification and characterization of the *GASA* family in *Lactuca sativa*

The identified 20 *GASAs* were renamed *LsGASA1* to *LsGASA20* according to their chromosomal locations and distributed across lettuce genome loci 1, 2, 3, 4, 8, and 9 (Additional file 1: Table S1).

The amino acid sequences of the 20 LsGASAs were analyzed, revealing that the sequences ranged from 78 to 662 amino acids in length, 8.34 to 67.73 kDa in molecular weight, 6.77 to 9.52 in isoelectric point, and − 0.33 to 0.21 in grand average of hydropathicity values (Additional file 1: Table S1). Prediction of subcellular localization indicated that most LsGASAs were found in the extracellular matrix, except for LsGASA4, LsGASA17, LsGASA19, LsGASA20, and LsGASA16, which are located in the chloroplast and cytoplasm, respectively (Additional file 2: Fig. S1).

**Fig. 1:**
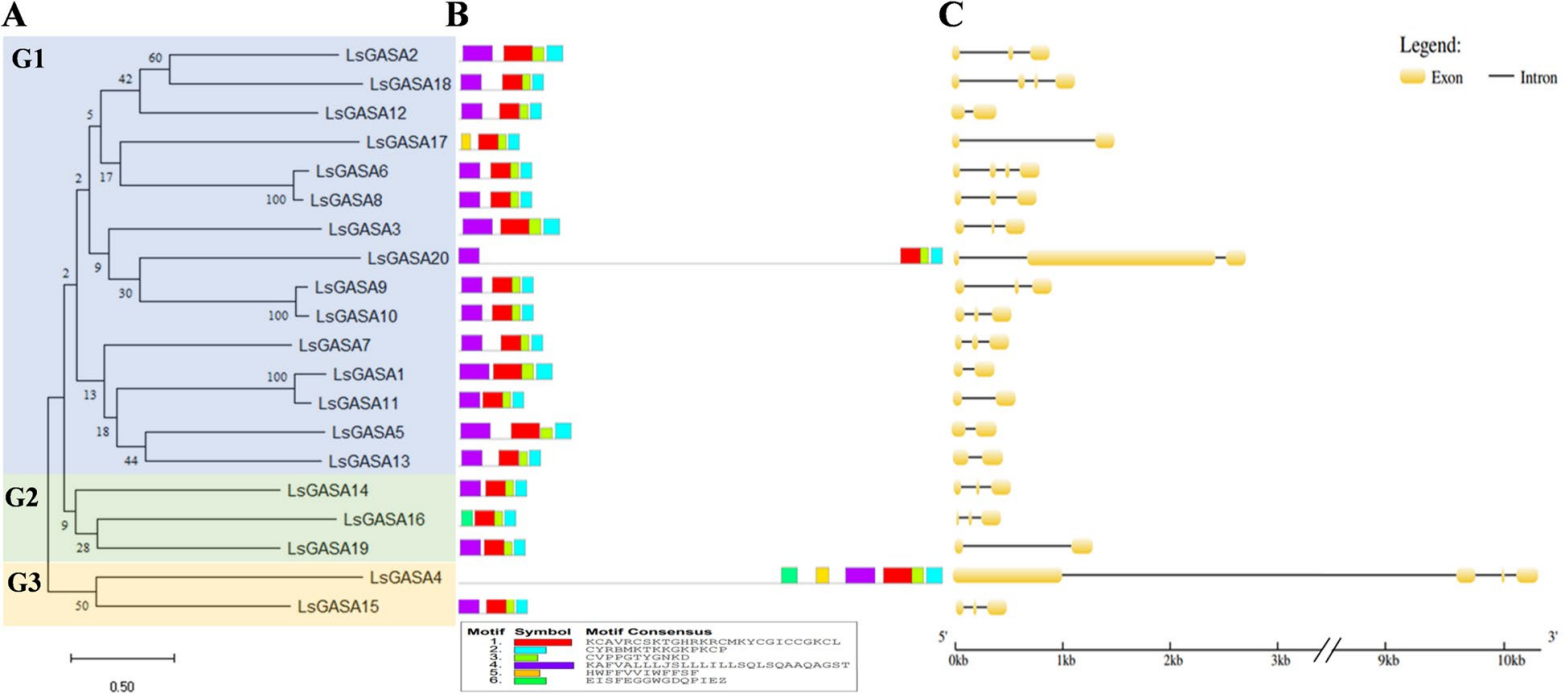
Phylogenetic analysis, conserved motifs of LsGASA proteins and exon-intron distribution. **A** Phylogenetic analysis of members of the GASA family in lettuce. **B** Conserved motifs in the LsGASA proteins. **C** Gene structure of *LsGASA*

A phylogenetic tree was constructed for the *LsGASA* (Fig. 1A), dividing it into three subclades. The exon-intron structure of the *LsGASA* contained no more than three introns (Fig. 1C). Most of the predicted motifs were distributed at the C-terminus of LsGASA proteins. One motif was present in the N-terminus of the LsGASA proteins; one motif was present except for LsGASA4 (Fig. 1B).

### Phylogenetic analysis and multiple sequence alignment

**Fig. 2:**
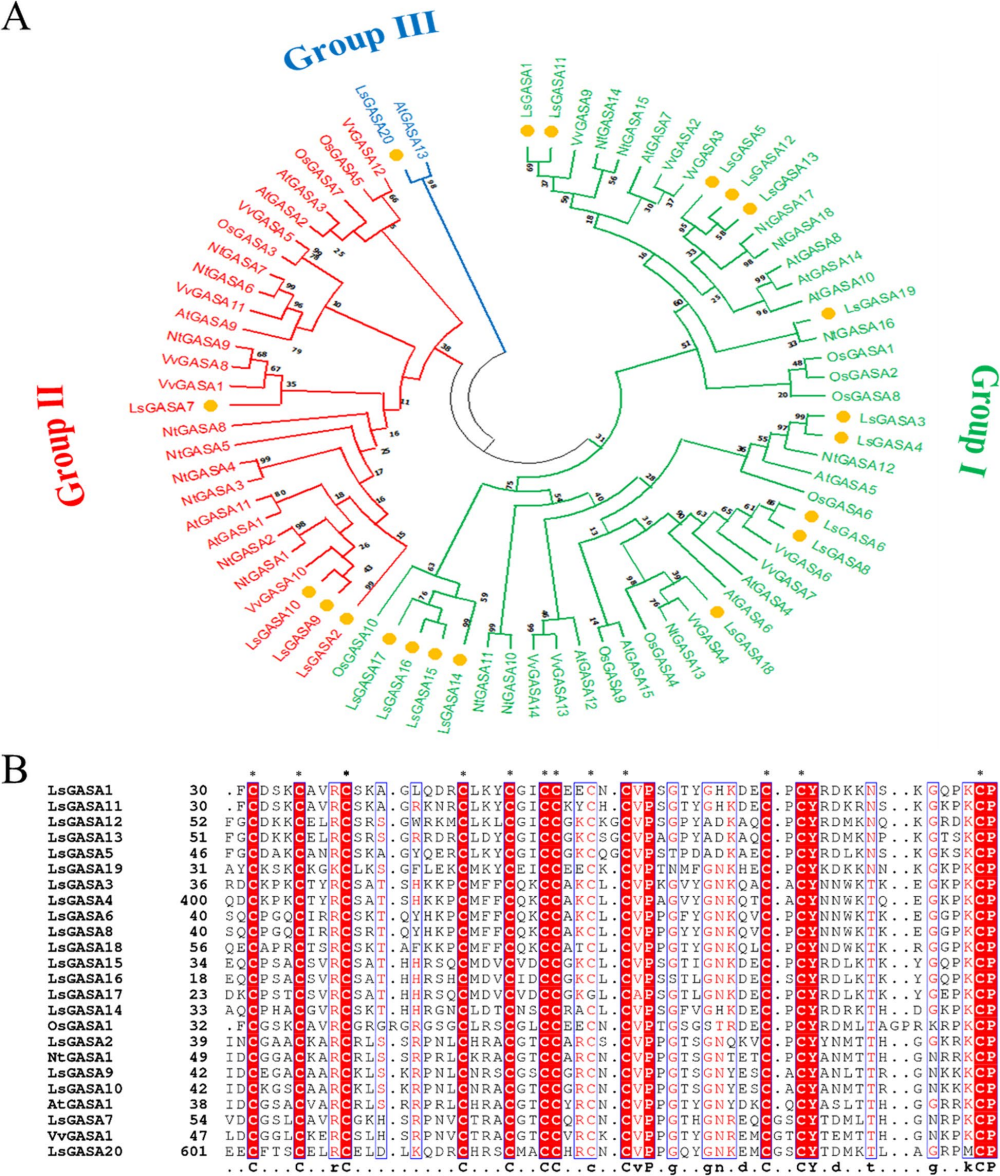
Phylogenetic analysis and amino acid sequence alignment from five plant species. **A** Phylogenetic tree of 77 *GASA* from lettuce (Ls: *Lactuca sativa* L.), Arabidopsis (At: *Arabidopsis thaliana* L*.*), grape (Vv: *Vitis vinifera* L.), rice (Oz: *Oryza sativa* L.), tobacco (Nt: *Nicotiana tabacum* L.). The different colors indicate different subfamily. **B** Sequence alignment of GASA proteins from LsGASA1–20, OsGASA1, NtGASA1, AtGASA1, and VvGASA1. Black asterisk represents their conserved cysteine region. Red background represented identical amino acids. Red text indicates similar sequences

Based on the phylogenetic analysis of *LsGASA*s with *GASA* families of other plant species, GASA protein families can be classified into three groups: Groups I, II, and III. In lettuce, Groups I, II, and III contained fifteen, four, and one LsGASA proteins, respectively (Fig. 2A). The amino acid sequence of the LsGASA proteins was compared to the GASA amino acid sequences of other species (Fig. 2B). Multiple alignments of the LsGASA amino acid sequences revealed the presence of a conserved GASA domain that included 12 cysteines in the C-terminal domain, except for LsGASA17.

### Chromosomal location, evolutionary relationships, and synteny analysis

**Fig. 3:**
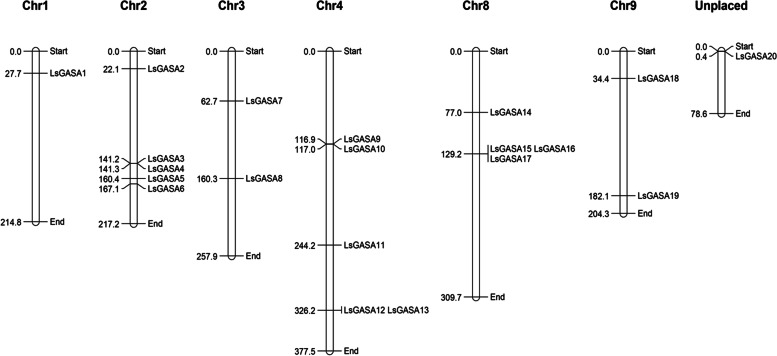
A chromosomal location of *GASA* in lettuce. The gene names are indicated on the right side of the chromosome, and physical locations (Mb) are indicated on the left side

Twenty *LsGASAs* were distributed on lettuce chromosomes 1 to 9, except for chromosomes 5, 6, and 7 (Fig. 3). Chromosomes 2, 4, and 8 contained more than three genes, whereas chromosomes 1, 3, and 9 contained fewer than three.

**Fig. 4:**
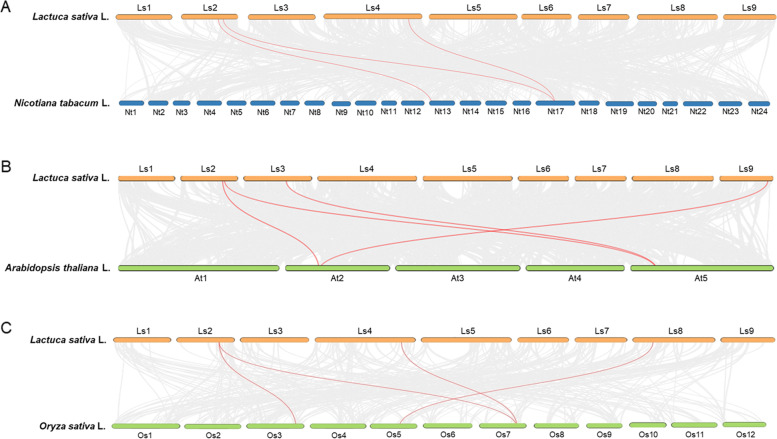
Synteny analysis of *Lactuca sativa* genome with model plants, (**A**) tobacco, (**B**) *Arabidopsis*, and (**C**) rice. The gray lines represent aligned blocks between the paired genomes, and the red lines indicate syntenic *GASA* pairs. Visualization was performed using Dual Synteny Plot in TBtools (v1.09876)

To deduce the evolutionary relationship of *GASA* among different species, syntenic analysis was performed for lettuce, tobacco, *Arabidopsis*, and rice (Fig. 4). Numerous syntenic blocks have been observed between lettuce and tobacco, *Arabidopsis*, and rice. Among the synteny blocks, three *LsGASA* genes (*LsGASA4*, *LsGASA5*, and *LsGASA12*) and four genes (*LsGASA5*, *LsGASA6*, *LsGASA8*, and *LsGASA19*) showed pairwise synteny with *NtGASA* and *AtGASA*, respectively. Furthermore, *LsGASA5*, *LsGASA12*, and *LsGASA14* showed pairwise synteny with the *OsGASA* family, with *LsGASA5* being syntenic with the both *OsGASA*.

In duplication analysis, six genes were tandemly duplicated and clustered by three duplication events on chromosomes 2, 4, and 8 (*LsGASA3/4*, *LsGASA12/13*, and *LsGASA15/16*). On the other hand, segmental duplications were detected on three gene pairs, including *LsGASA1/11*, *LsGASA6/8*, and *LsGASA9/10* (Additional file 3: Table S2). The Ka/Ks ratio was determined using tandem-and segmentally duplicated gene pairs. Tandem and segmentally duplicated gene pairs showed Ka/Ks < 0.6, and events were estimated to be between 5.4 and 39.3 Mya (Additional file 3: Table S2).

### Determination of cis-regulatory elements in the promoters of *LsGASA*

**Fig. 5:**
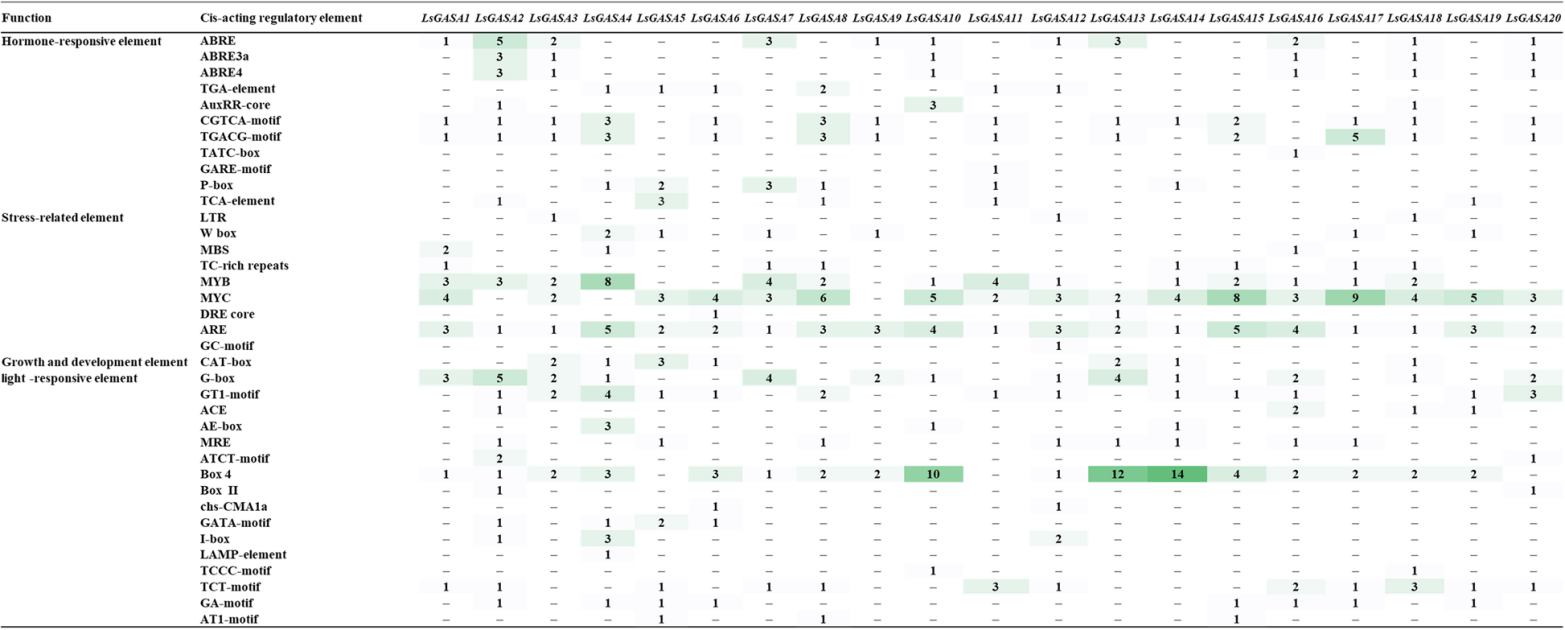
Number of each cis-acting element of the *LsGASA* promoter regions (1.5 kb upstream of the start codon). ABRE, ABRE3a, ABRE4: abscisic acid responsiveness. AuxRR-core: auxin responsiveness. CGTCA-motif, TGACG-motif: MeJA responsiveness. TATC-box, GARE-motif, P-box: gibberellin-responsive elements. TCA-element: salicylic acid responsiveness elements. LTR: involved in low-temperature responsiveness. W box: involved in both biotic and abiotic stress responses. MBS: drought inducibility. TC-rich repeats: involved in defense and stress responsiveness. MYB, MYC: ABA- and drought-responsive elements. DRE core: dehydration-responsiveness. ARE: essential for anaerobic induction. GC-motif: hypoxia-induced response element. CAT-box: related to meristem expression. G-box, GT1-motif, ACE: involved in light responsiveness. AE-box: part of the module for light response. MRE: MYB-binding site involved in light responsiveness. ATCT-motif, Box 4: part of a light responsive module. Box 2, chs-CMA1a, GATA-motif, I-box, LAMP-element, TCCC-motif, TCT-motif, GA-motif: part of a light-responsive element. AT1-motif: part of a light-responsive module

To investigate the potential roles of *cis*-acting elements in *LsGASA*s, we analyzed the sequences 1.5 kb upstream of each *LsGASA*. The promoter region of the LsGASA genes contained hormone-responsive, stress-related regulatory, growth-, developmental-, and light-responsive elements (Fig. 5). The *cis*-elements that responded to hormones included abscisic acid response elements (ABREs), auxin-responsive elements (TGAelement and AuxRR-core), gibberellin-responsive elements (TATC-box, P-box, and GARE-motif), and MeJA response elements (TGACG-motif and CGTCA-motif). Except for *GASA19*, all other *GASA*s contained at least one sequence of hormone-responsive *cis*-acting elements. *LsGASA*s contain various stress-related elements, including ARE, DRE core, GC motif, LTR, MBS, MYB, MYC, and TC-rich repeats. Most *GASA* contains 35 MYB, 70 MYC, and 48 ARE elements in their promoters. All *GASA* contained ARE elements. In the promoter of *LsGASA*s, light-responsive elements (ACE, AE-box, AT1-motif, ATCT-motif, Box 4, Box II, G-box, chs-CMA1a, GA-motif, GT1-motif, GATA-motif, I-box, LAMP-element, MRE, TCCC-motif, and TCT-motif) showed the largest number compared to hormone-responsive and stress-responsive elements. Among the various elements, all *GASA* contained Box 4 elements that were associated with light-responsive elements (Fig. 5).

### Expression profiling of *LsGASA* in responses to heat stress in lettuce SAM

**Fig. 6:**
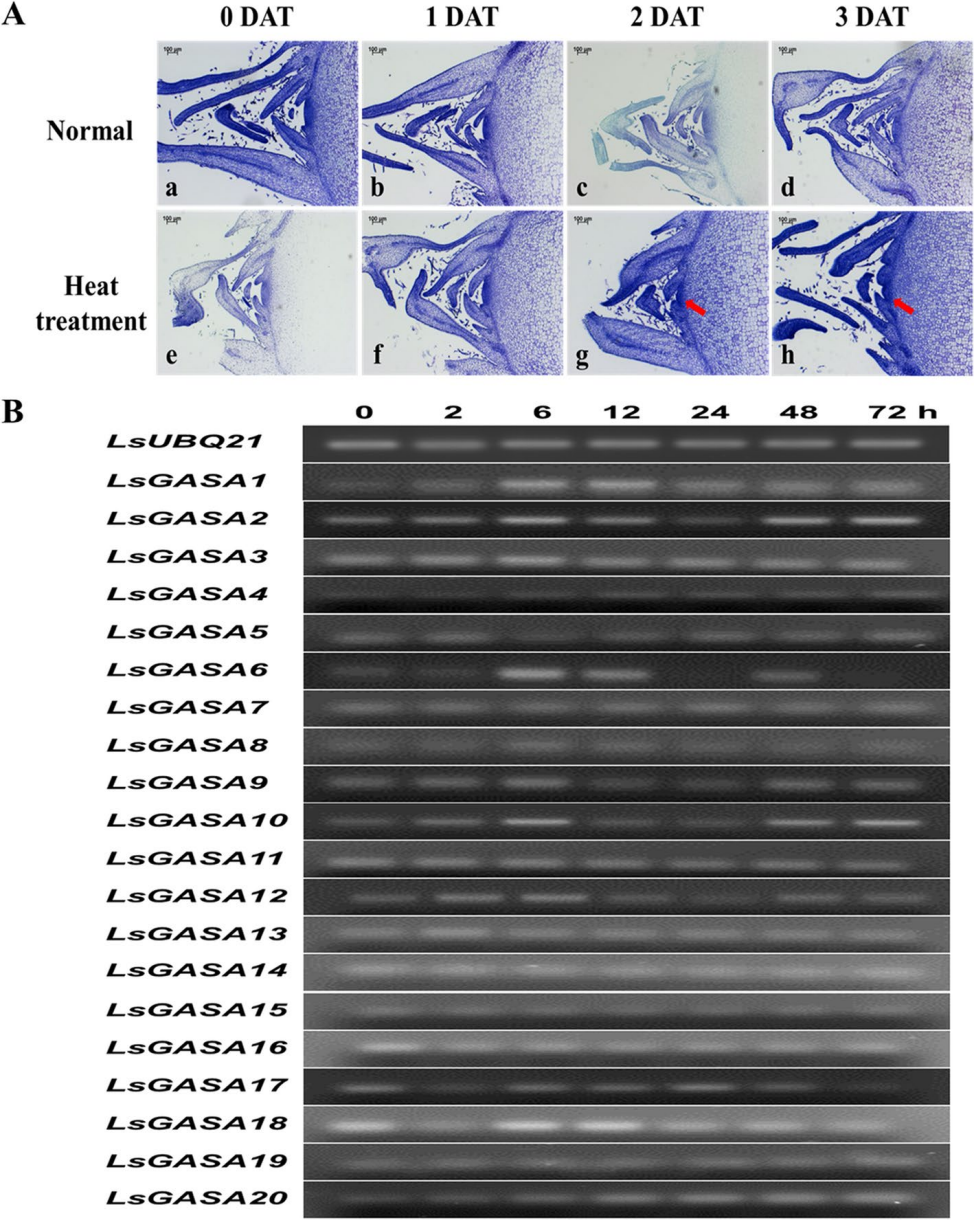
Paraffin sections and semi qRT-PCR analysis in shoot apical meristem (SAM) under heat stress conditions. **A** paraffin sections of SAM under high temperature conditions of 35/25 °C for 3 days.(a–e) control, (g–h) heat treatment. Toluidine Blue O stained tissues (Sections = 8 μm, Bars = 100 μm). DAP; Days after treatment. **B** Semi qRT-PCR analysis of 20 *LsGASA* in SAM under high temperature conditions of 35/25 °C for 72 h (uncropped full length gel image is provided in the Supplementary Information file Fig. S1)

To examine the effect of heat stress on lettuce SAM development, the morphological features of lettuce SAM were observed (Fig. 6A). There was no noticeable difference between days 0 and 1 in the control or heat stress treatments. On day 2, the heat-stressed plants had a larger dome size than the control plants. On day 3 of the heat stress treatment, the shoot apical meristem became sharper. These results suggest that lettuce bolting is promoted by high temperatures.

**Fig. 7:**
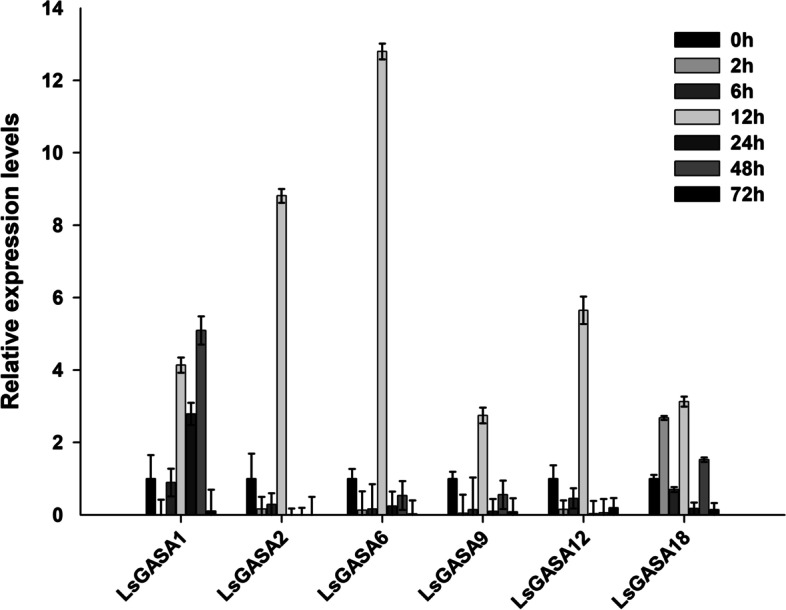
Expression analysis of six lettuce *GASA* in shoot apical meristem (SAM) response to heat stress conditions of 35/25 °C for 72 h. Error bars indicate standard errors

To investigate the response of *LsGASA* in the SAM of lettuce under heat stress, we performed semi-qRT-PCR at seven different time points: 0, 2, 6, 12, 24, 48, and 72 h of heat treatment (Fig. 6B). The band pixel intensities of *LsGASA*s and *LsUBQ21* were quantified using ImageJ, presenting a computerized method to express the degree of gene expression (Additional file 4: Fig. S2). Similar expression patterns were observed for *LsGASA1* and *LsGASA18*, which peaked at 6 h and subsequently gradually decreased after 24 h of heat treatment (Fig. 6B). The expression levels of *LsGASA2*, *LsGASA9*, and *LsGASA12* gradually increased until 6 h and then decreased. However, *LsGASA2*, *LsGASA9*, and *LsGASA12* expression increased again from 48 h to 72 h of heat treatment. Transcripts of *LsGASA6* were highly increased until 6 h and then decreased but increased again after 48 h of heat treatment (Fig. 6B). Transcripts of *LsGASA2*, *LsGASA6*, *LsGASA9*, and *LsGASA12* exhibited similar expression patterns in qRT-PCR, peaking at 12 h and then decreasing (Fig. 7). *LsGASA1* expression increased from 12 to 48 h. *LaGASA18* was highly expressed after 12 h of heat treatment.

For further study, we selected six *LsGASA* (1, 2, 6, 9, 12, and 18) that displayed increased expression in the SAM of lettuce under heat treatment. These results suggest that the six *LsGASA*s may be involved in heat-induced bolting mechanisms.

### Expression analysis of six *LsGASA* in response to abiotic stresses and hormone treatments

To evaluate the expression levels of the six selected *LsGASA*s across an array of lettuce tissue samples, we analyzed the expression of the six *GASAs* in leaves, roots, stems, seeds, and flowers (Additional file 5: Fig. S3). *LsGASA2*, *LsGASA6*, *LsGASA9*, and *LsGASA12* were more highly expressed in flower buds than in other tissues. *LsGASA18* transcripts were highly expressed in seeds. *LsGASA1* was highly expressed in the stems and roots compared to other tissues.

**Fig. 8:**
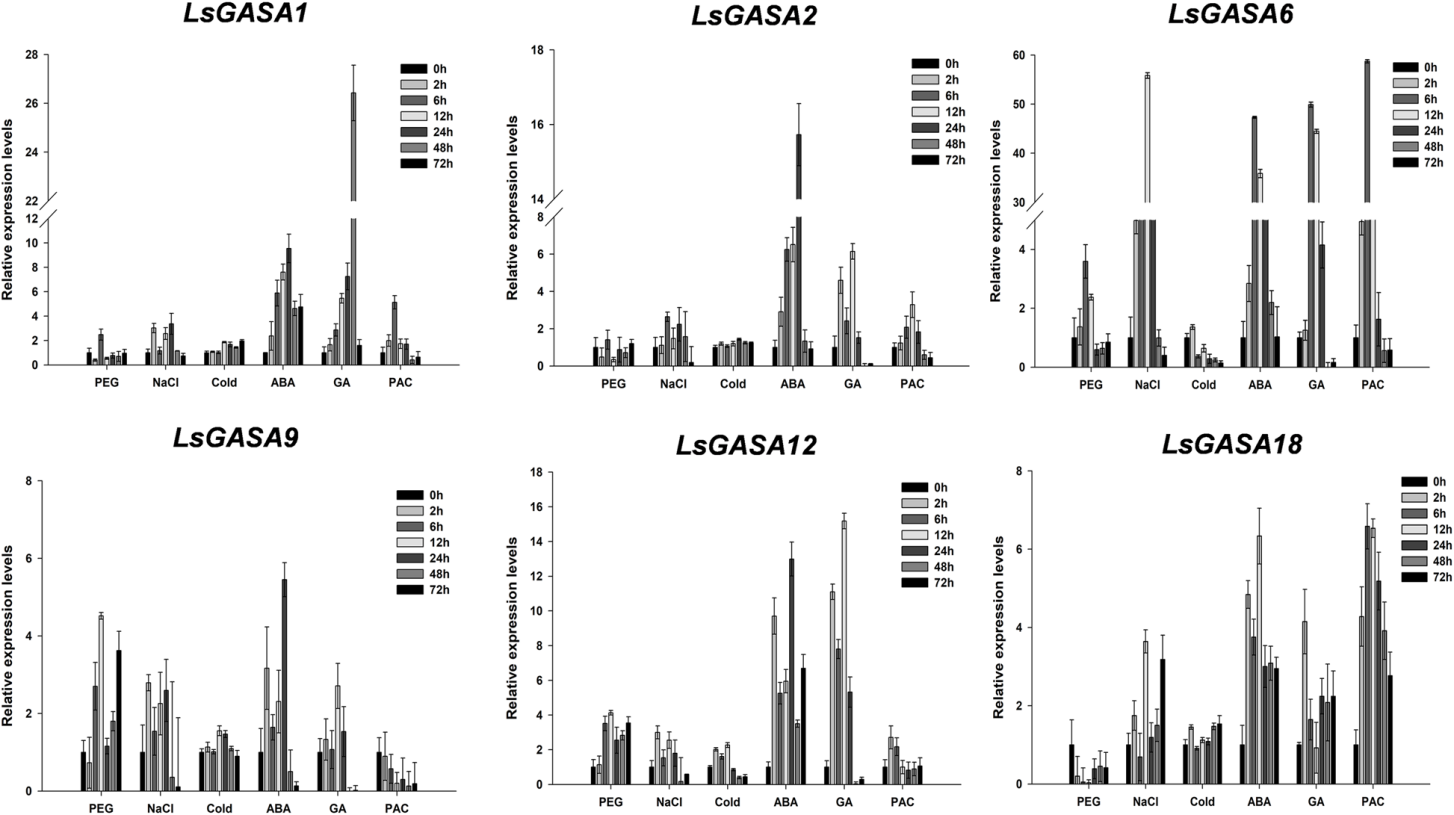
Expression analysis of 20 *LsGASA* in response to stress and hormone treatments. 20% polyethylene glycol (PEG), 250 mM sodium chloride (NaCl), Cold (4 °C), 100 μM abscisic acid (ABA), 150 μΜ gibberellin acid (GA_3_), 150 μΜ paclobutrazol (PAC). All treatments were conducted for 72 h. Error bars are the standard error of the mean

To investigate the effects of abiotic stresses and hormones on the expression of the six *LsGASA*, different stimuli, including PEG, NaCl, low temperatures, GA, PAC, and ABA, were applied (Fig. 8). Under PEG treatment, *LsGASA1*, *LsGASA6*, and *LsGASA9* showed similar expression patterns, increasing at 6 h and decreasing at 12 h of heat treatment. The transcript levels of *LsGASA12* increased until 12 h and then decreased at 24 h. The expression of *LsGASA2* and *LsGASA18* decreased after treatment. In the NaCl treatment, all *GASA* genes showed increased expression levels. In particular, *LsGASA6* was highly expressed after NaCl treatment compared to other *GASAs*. Under cold treatment, *LsGASA1* and *LsGASA12* showed increased expression. By contrast, the expression levels of *LsGASA2*, *LsGASA6*, *LsGASA9*, and *LsGASA18* were not significantly different. Following GA treatment, all *GASA*s showed upregulated expression. The transcript of *LsGASA1* gradually increased from 6 h to 48 h. *LsGASA6* showed maximum expression levels at 6 h, whereas *LsGASA2*, *LsGASA9*, and *LsGASA12* showed high expression levels at 12 h. Under ABA treatment, all *GASA* genes showed upregulated expression, and *LsGASA6* showed the highest expression under ABA treatment. Following PAC treatment, the expression levels of all *LsGASA* showed upregulation patterns, which increased at 6 h, except for *LsGASA9*.

### Subcellular localization of *LsGASA*

**Fig. 9:**
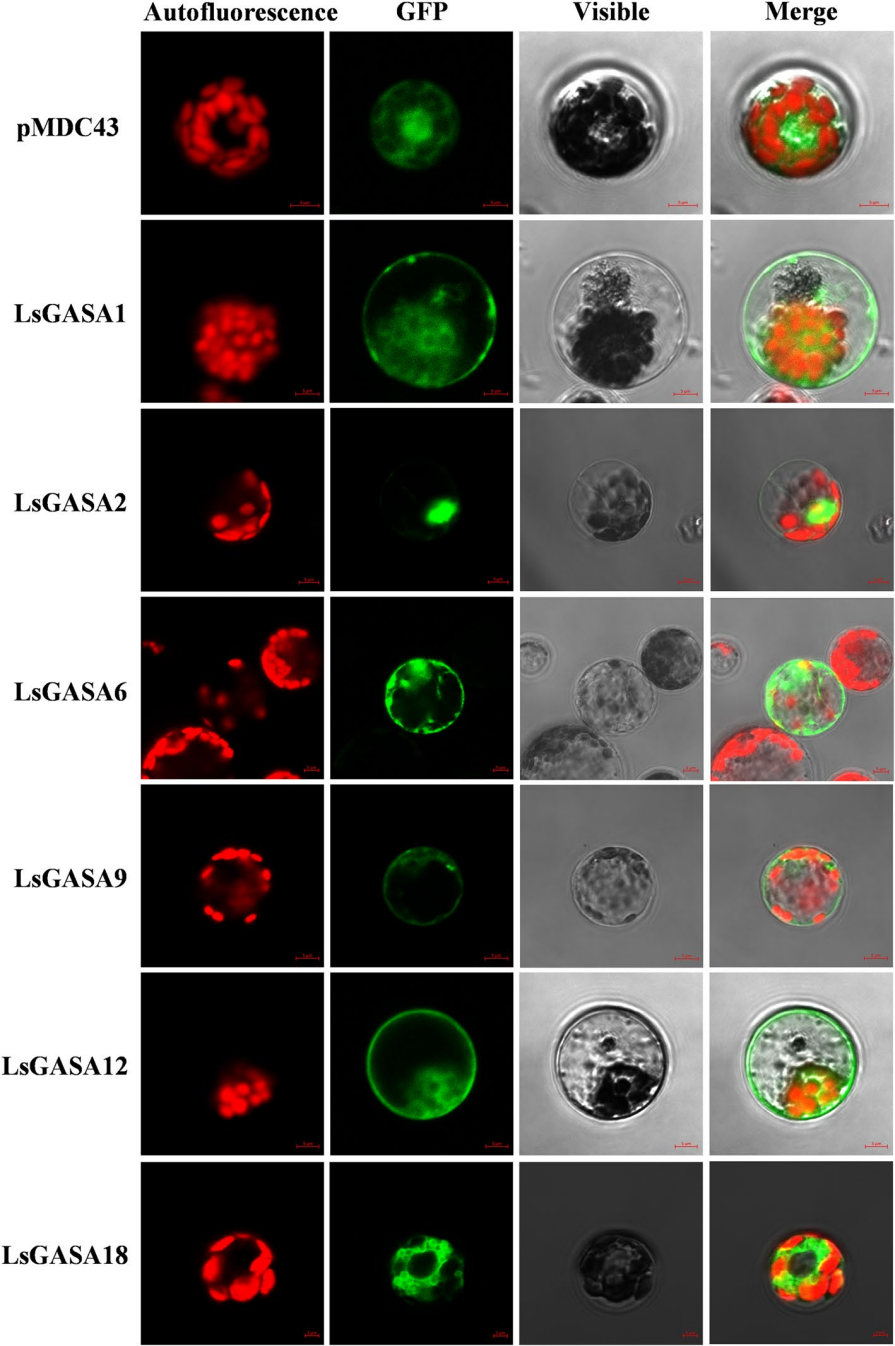
Subcellular localization of six LsGASA proteins in lettuce protoplast (Scale bar = 5 μm)

To examine the subcellular localization of the six LsGASAs, cassettes of LsGASA-GFP were transfected into lettuce protoplasts. Most LsGASA-GFP fusion proteins have been detected in the plasma membrane, cytoplasm, or both. LsGASA1-GFP, LsGASA9-GFP, and LsGASA18-GFP were localized to the cytoplasm. LsGASA6-GFP and LsGASA12-GFP are located in the plasma membrane and cytoplasm, respectively. However, only the LsGASA2-GFP fusion protein was detected in the nucleus (Fig. 9).

## Discussions

GASA proteins are essential for hormone signaling, abiotic and biotic stress responses, and many aspects of plant growth and development [9, 18, 29, 30]. However, studies on the role of *GASA* in high-temperature-induced lettuce bolting are lacking. In this study, we identified 20 *GASA* in the lettuce genome, and six *GASA* that were highly expressed in SAM under heat stress were chosen and further characterized. Most of the amino acid sequences of the LsGASA proteins ranged in length from 78 to 116 amino acids (Additional file 1: Table S1). However, the lengths of the amino acid sequences of LsGASA4 and LsGASA20 were 460 aa and 662 aa, respectively. The cDNA lengths of the *LsGASA*s were also similar, except for *those of LsGASA4* and *LsGASA20* (Additional file 1: Fig. S1 and Fig. 1C). Protein domain analysis revealed that they contained a signal peptide, except for *LsGASA16* and *LsGASA17* (Fig. 1B). Furthermore, the number of exons and introns in the gene structure analysis varied from one to three (Fig. 1C). These results indicate that *LsGASA* may gain or lose exons or introns because of chromosomal rearrangements [31].

Phylogenetic analysis of *LsGASA* along with other *GASA* revealed a closer relationship with dicotyledonous species than with monocotyledonous species (Fig. 2A). Gene duplication analysis of the *LsGASA* family revealed three tandem duplications and five segmental duplication gene pairs (Additional file 3: Table S2). These findings are consistent with a previous report indicating that segmental duplications occur more frequently than tandem duplications [7]. Evolutionary events related to duplication can affect the structure of the genome. Gene structure analysis revealed that the *LsGASA* family varied in terms of exons (Fig. 1C). However, there was no significant difference in the number of exons among the three subgroups (Fig. 2). The number of exons can influence post-transcriptional processes such as alternative splicing. The number of exons can also affect the expression patterns of genes, specifically resulting in genes with fewer exons being quickly induced [32]. Gene expression analysis revealed that *LsGASA*s exhibited a variety of expression patterns in response to abiotic stress and phytohormones (Fig. 8). The *VvGASA* family is mainly distributed on one chromosome; on the other hand, the *LsGASA* family is widely distributed on three chromosomes (Fig. 3). These results suggest that duplication of *GASA* occurs on multiple chromosomes during the expansion of the lettuce *GASA* family. From the synteny analysis, it was shown that less collinearity was observed between lettuce and rice compared to the other two dicot species (Fig. 4), which is thought to be because lettuce and rice separated earlier in the past. Furthermore, different types of *LsGASA* were collinear with *GASA*s in other species, whereas only *LsGASA5* homologs were found in all species. This suggests that *GASA5* is extensively conserved across plant species and is important for plant growth.

*Cis*-element analysis of the *LsGASA* showed that most *LsGASA*s contained various hormone-responsive elements in their promoters (Fig. 5). In the promoters of *LsGASA*s, ABA-responsive ABRE elements, auxin-responsive CGTCA, and MeJA-responsive TGACG motifs were abundant (Fig. 5). Previous studies have revealed that the transcript of *GASA* from *Populus euphratica* is downregulated in leaves under MeJA treatment [33]. In *Prunus mume,* six *GASA* were upregulated after ABA treatment, and five *GASA* were upregulated at different time points after IAA treatment [34]. In the present study, all the *LsGASA*s contained at least four regulatory elements related to light-responsive elements in their promoters (Fig. 5). Light has also been reported to affect AtGASA expression [13]. *AtGASA4* transcripts are downregulated by far-red light signaling regulators under light conditions, resulting in early flowering [35]. Additionally, *LsGASA* contained several MYB and MYC elements (Fig. 5). The *XsMYB44* transcription factor plays a positive regulatory role in response to combined stresses by inducing stomatal closure and regulating ROS homeostasis [36]. The presence of multiple regulatory elements in *LsGASA* promoters indicates that *GASA* may participate in various hormone-signaling pathways.

In general, high temperatures promote bolting in lettuce plants. In a previous study, transcripts of *LsGA3ox1* were not only upregulated by high temperatures but also increased by GA, leading to bolting [37]. In our study, the development process of SAM revealed that high-temperature-treated plants increased the number of bolts faster than control plants (Fig. 6A). Furthermore, the transcript levels of *LsGASA*s (*LsGASA1*, *LsGASA2*, *LsGASA6*, *LsGASA9*, *LsGASA12*, and *LsGASA18*) in SAM were highly increased under heat treatment (Fig. 7). These findings imply that these six *GASAs* may be involved in the heat-induced bolting pathway.

The *GASA* family has been reported to be differentially expressed in different organs as well as in diverse abiotic stresses and hormones [4, 38, 39]. Expression analysis of lettuce organs showed that six *LsGASA* were expressed in the leaves, roots, seeds, stems, and flowers (Additional file 5: Fig. S3). Most of these genes were upregulated in roots, seeds, and flowers. In particular, *LsGASA6* was most strongly expressed in the flowers (Additional file 5: Fig. S3). Previous studies have reported that *NtGASAs* and *MdGASAs* are more highly expressed in flowers and buds than in other organs [8, 38]. In addition, the transcripts of six *LsGASA*s showed an increasing pattern in the cold, PEG, and NaCl treatments (Fig. 8). Previous studies have reported that *GASA* is involved in various abiotic stressors and hormones. Overexpression of *GASR1* increases salt stress tolerance in transgenic rice [40]. *SmGASA4*-overexpression in *Arabidopsis* promotes drought resistance [41]. *AtGASA4* and *AtGASA6* are upregulated by growth hormones and downregulated by stress hormones [15]. In this study, six *LsGASA* were highly expressed in response to ABA and GA. After PAC treatment, the expression levels of *LsGASA*s increased, except for *LsGASA9* (Fig. 8). Based on the above evidence, our results suggest that the six *GASA* may play diverse roles related to abiotic stresses as well as hormone-induced bolting and flowering pathways.

According to subcellular localization analyses in various crops, GASA proteins are mostly located in the extracellular region and plasma membrane [14, 16, 42]. In the present study, the subcellular location of most LsGASA proteins was predicted to be in the extracellular regions (Additional file 2: Fig. S1). However, the six selected LsGASA proteins were mostly localized in the plasma membrane or cytoplasm of lettuce protoplasts, or both (Fig. 9). A previous study showed that *CcGASA4*, which is highly expressed in hormone-treated flowers, is localized in the plasma membrane and nucleus in *Arabidopsis* protoplasts [42]. *GhGASA10–1* from cotton, which is localized in the cell membrane of tobacco epidermal cells, is highly expressed by IAA during the fiber elongation stages [14]. LsGASA6 and LsGASA12 proteins were localized in the plasma membrane and cytoplasm, and LsGASA2 was localized in the nucleus (Fig. 9). They were highly expressed in flowers as well as in the hormone treatments (Additional file 5: Fig. S3 and Fig. 8). These results suggest that *LsGASAs* may participate in various hormonal signaling pathways and plant developmental processes.

## Conclusions

In this study, we identified 20 *LsGASA* families in the lettuce genome. Sequence analysis revealed that *LsGASA* contains a conserved GASA domain, and the distributions of exons and introns were different among the different subgroups. The *LsGASA*s were located on six chromosomes and were distributed into three groups according to phylogenetic analysis. In the analysis of *cis*-regulatory elements, *LsGASA*s contain various stress-, light-, and hormone-related elements. We analyzed the expression patterns of 20 *GASA* genes in SAM under heat stress and found six genes predicted to be related to heat-induced bolting. Six *LsGASA* (*LsGASA1*, *LsGASA2*, *LsGASA6*, *LsGASA9*, *LsGASA12*, and *LsGASA18*), which are heat-induced genes in SAM, were investigated for their expression patterns in different organs and response to abiotic stress and hormones. Tissue-specific expression revealed that *LsGASA*s were highly expressed in the roots and flowers. Six *LsGASA*s exhibited diverse expression patterns in response to different abiotic stressors and hormone treatments. In addition, the subcellular localization of the six LsGASAs was mostly in the plasma membrane, cytoplasm, or both. Overall, this study provides fundamental information on the *LsGASA* family and its responses to various abiotic stresses. This study will allow for a comprehensive investigation into the functional roles of each *LsGASA*; these findings will then allow for future studies into the characterization of mechanisms that underlie heat-induced bolting in lettuce.

## Materials and methods

### Identification of the *LsGASA* family in lettuce

To identify *LsGASA*, the hidden Markov model (HMM) profiles of the GASA domain (PF02704) from the Pfam database (https://pfam.xfam.org/) were used as a query, and the putative GASA protein sequences were identified using HMMER v 3.0 [43] searching against the lettuce genome (https://plants.ensembl.org/index.html) with a predefined threshold of E < 1e-5. The selected proteins containing GASA domains were confirmed using InterProScan (https://www.ebi.ac.uk/interpro/search/sequence-search). The ExPASy ProtParam (https://web.expasy.org/protparam/) tool was used to estimate the physical and chemical characteristics of all identified GASA proteins, including their isoelectric point, molecular weight, and grand average of hydropathy.

### Chromosome distribution and evolutionary analysis of *LsGASA*

The chromosomal localization of *LsGASAs* was determined using the lettuce genome database. The MapChart program was used to graphically map lettuce chromosomes [44]. The CDSs of *LsGASA* were aligned using ClustalW and KaKs_calculator 3.0 [45] was used to determine the rates of non-synonymous (Ka) and synonymous (Ks) substitutions for duplicate *LsGASA* pairs. The divergence time (*T*) was calculated using the following eq. [46]:

1 $$T= Ks/2x$$

where *x* = 6.56 × 10^− 9^.

TBtools [47] was used to identify syntenic relationships between lettuce and the tobacco/*Arabidopsis* genome. GFF files were obtained from EnsemblPlants (https://plants.ensembl.org/index.html) for lettuce and *Arabidopsis*, and from the Sol Genomics Network (https://solgenomics.net/organism/Nicotiana_tabacum/genome) for tobacco. The genome sequences and GFF files of lettuce and other species were used as input files for the One-Step MCScanX tool in TBtools. Consequently, the output files, Ctl, simplified GFF, and collinearity file were used for the dual synteny plot in TBtools for synteny visualization.

### Phylogenetic tree, multiple alignments, and gene structure analysis

A phylogenetic tree was constructed using the neighbor-joining method with 1000 bootstrap replicates using MEGA X software [48]. Multiple sequence alignment of GASA proteins was performed using ESPript 3.0 [49]. The Gene Structure Display Server 2.0 (https://gsds.gao-lab.org/) was used to display the exon-intron structures of the *LsGASA*.

### Conserved motif and *cis*-acting regulatory elements analysis

The conserved motifs of LsGASA proteins were predicted using MEME Suite (http://meme-suite.org/), and the subcellular localization of LsGASA proteins was predicted using WoLF PSORT (https://wolfpsort.hgc.jp/) and Plant-mPLoc server (https://rostlab.org/services/nlsdb/). Furthermore, the *cis*-acting elements of the promoters up to 1500 bp upstream of the start codon of all *LsGASA*s were predicted using PlantCARE (http://bioinformatics.psb.ugent.be/webtools/plantcare/html/).

### Subcellular localization analysis

To examine the subcellular localization of LsGASAs, the CDS of *LsGASA* were cloned into the pCR/GW/TOPO cloning vector (Invitrogen, Carlsbad, CA, USA) and then cloned into the pMDC43 vector using LR Clonase (Invitrogen, Carlsbad, CA, USA). Recombinant vectors containing the GFP expression cassette were examined for transient expression using lettuce protoplast transfection. The subcellular localization of LsGASAs was observed using a confocal laser scanning microscope (LSM 700, Carl Zeiss, Jena, Germany).

### Plant material, growth, and abiotic stress conditions

The lettuce cultivar *D*. *atrakce* (accession no. IT275492; National Agrobiodiversity Center, Korea) was used in this study. Germplasm was provided by the National Agrobiodiversity Center, Korea, with permission for use in this experiment. *D*. *atrakce* was grown in 32 cell trays and placed in a growth chamber (HB-301 L-3, Hanbaek science, Korea) set at 22/20 °C (16 h/8 h), 60% relative humidity, and 18,000 lx light intensity. Plants were exposed to abiotic stress and hormone treatment 25 d after planting. To examine the responses of the *LsGASA*s to abiotic stresses and phytohormone applications, 25-day-old seedlings were subjected to diverse treatments such as heat stress (35/25 °C, 16 h/8 h), 20% polyethylene glycol (PEG 6000), 250 mM sodium chloride (NaCl), low temperature (4 °C), 150 μΜ of GA_3_, 150 μΜ of paclobutrazol (PAC) and 100 μM of ABA, and the treated lettuce seedling shoot apical meristem (SAM) and leaves were harvested at time intervals of 2, 6, 12, 24, 48 and 72 h. All the obtained samples were immediately stored at − 80 °C until further use.

### Paraffin sections

SAM of heat-stressed ‘*Detenicka atrakce’* was fixed with 4% paraformaldehyde, followed by vacuum for 20 min. An ethanol series of 30, 50, 70, 95, and 100% was used to dehydrate the fixed samples. The dehydrated samples were cleaned with *tert*-butyl alcohol (Sigma-Aldrich, USA) series (35, 50, 70, and 100%). The cleaned samples were infiltrated and embedded in Paraplast Plus (Sigma-Aldrich) at 58 °C. Embedded samples were cut into 8-μm thick sections using a microtome (Leica RM2255). For observation, sectioned samples were stained with 0.05% toluidine blue O (Sigma-Aldrich) in citrate buffer (pH 4) and coverslips were applied using Canada balsam (Duksan Science, Korea). Finally, slides were observed under an upright microscope (Carl Zeiss, Jena, Germany).

### Gene expression analysis

Total RNA was extracted from organ tissues (leaves, roots, stems, seeds, and flowers) and the extract was treated with TRIzol reagent (Invitrogen, Carlsbad, CA, USA). A Power cDNA synthesis kit (iNtRON Biotechnology, Korea) was used to synthesize first-strand circular DNA (cDNA) from 1 μg total RNA according to the manufacturer’s instructions. Polymerase chain reaction (PCR) was used to confirm the specificity of gene-specific primers designed using Primer-BLAST (https://www.ncbi.nlm.nih.gov/tools/primer-blast/index.cgi? LINK_LOC=BlastHome) (Additional file 6: Table S3). Diluted cDNAs were used as templates for semi-quantitative real-time (RT)-PCR analysis. Semi-qRT-PCR was performed as follows: initial denaturation at 95 °C for 5 min, followed by 35 cycles of 1 min at 95 °C, 30 s at 58 °C, 1 min at 72 °C, and a final elongation step at 72 °C for 5 min. Quantitative real-time PCR (qRT-PCR) was performed using EvaGreen 2X qPCR Mastermix (ABM, Vancouver, BC, Canada) on a CFX-96 RT-PCR systems (Bio-Rad Laboratories Inc., Hercules, CA, USA) with three independent replicates. Gene expression was calculated as the fold change using the 2^−ΔΔCT^ method [50].

## Supplementary Information

**Additional file 1: Table S1.** Characteristics of LsGASAs from lettuce.

**Additional file 2: Fig. S1.** A heat map of predicted subcellular localization of 20 LsGASA family. Nucl: nucleus, Cyto: cytoplasm, Mito: mitochondria, Cysk: cytoskeleton, Chlo: chloroplast, E.R: endoplasmic reticulum, Plas: plasma membrane, Golg: golgi apparatus, Pero: peroxisome, and Extra: extracellular.

**Additional file 3: Table S2.** Ks, Ka, and Ka/Ks calculation and divergent time of the duplicated LsGASA pairs.

**Additional file 4: Fig. S2.** Semi qRT-PCR analysis of 20 LsGASA in shoot apical meristem (SAM) under heat stress conditions using ImageJ program.

**Additional file 5: Fig. S3.** Expression analysis of six LsGASA in different tissues (leaf, root, seed, stem and flower). Error bars represent the standard error of the mean.

**Additional file 6: Table S3.** List of specific primers used in the study.

## Data Availability

The genome sequences of *Lactuca Sativa* L. were downloaded from the Phytozome database (https://phytozome-next.jgi.doe.gov/info/Lsativa_V8) and the datasets supporting the conclusions of this article are included in the article and its Additional files.
