# Supplementary material for: Genome-wide identification and characterization of the lettuce GASA family in response to abiotic stresses
Source: BMC Plant Biol. 2023 Feb 22;23:106. doi: 10.1186/s12870-023-04101-5 (PMC9945619; doi:10.1186/s12870-023-04101-5)
Supplement: Supplementary file 1 — Additional file 1: Table S1. Characteristics of LsGASAs from lettuce. [file 12870_2023_4101_MOESM1_ESM.docx]

**Table S1.** Characteristics of LsGASAs from lettuce.

| **Gene name** | **Gene ID (Last_Salinas_V7)** | **Chromosome** | **location** | **cDNA length** | **Peptide length** | **MW^z^ (kDa)** | **PI^Y^** | **GRAVY^x^ (Grand average of hydropathicity)** |
| --- | --- | --- | --- | --- | --- | --- | --- | --- |
| *GASA1_qRT* | LSAT_1X23380 | 1 | 27679568-27680185 | 270 | 89 | 9.73 | 8.46 | -0.1337 |
| *GASA2_qRT* | LSAT_2X9381 | 2 | 22105104-22106306 | 300 | 99 | 10.56 | 8.36 | 0.0202 |
| *GASA3_qRT* | LSAT_2X69401 | 2 | 141229218-141230306 | 291 | 96 | 10.67 | 9.52 | -0.1677 |
| *GASA4_qRT* | LSAT_2X69421 | 2 | 141255929-141266454 | 1383 | 460 | 51.48 | 9.16 | -0.4628 |
| *GASA5_qRT* | LSAT_2X84540 | 2 | 160409772-160410356 | 324 | 107 | 11.13 | 8.62 | -0.0617 |
| *GASA6_qRT* | LSAT_2X90361 | 2 | 167125163-167126075 | 303 | 100 | 10.76 | 9.29 | 0.023 |
| *GASA7_qRT* | LSAT_3X49620 | 3 | 62702503-62703468 | 348 | 115 | 12.31 | 8.94 | -0.0904 |
| *GASA8_qRT* | LSAT_3X101621 | 3 | 160275983-160276746 | 303 | 100 | 11.03 | 9.44 | -0.221 |
| *GASA9_qRT* | LSAT_4X74100 | 4 | 116883306-116884207 | 309 | 102 | 10.98 | 8.77 | -0.2647 |
| *GASA10_qRT* | LSAT_4X74180 | 4 | 116993606-116994131 | 309 | 102 | 11.04 | 9.11 | -0.2569 |
| *GASA11_qRT* | LSAT_4X129301 | 4 | 244186876-244187459 | 270 | 89 | 9.75 | 9.35 | -0.1315 |
| *GASA12_qRT* | LSAT_4X162200 | 4 | 326193975-326194395 | 342 | 113 | 12.11 | 8.87 | -0.1248 |
| *GASA13_qRT* | LSAT_4X162220 | 4 | 326196970-326197796 | 339 | 112 | 11.57 | 9.1 | 0.0545 |
| *GASA14_qRT* | LSAT_8X55660 | 8 | 76995009-76995540 | 282 | 93 | 10.12 | 8.45 | -0.1215 |
| *GASA15_qRT* | LSAT_8X88640 | 8 | 129159237-129159710 | 285 | 94 | 10.05 | 7.44 | 0.2064 |
| *GASA16_qRT* | LSAT_8X88660 | 8 | 129187574-129187988 | 237 | 78 | 8.34 | 6.77 | -0.0654 |
| *GASA17_qRT* | LSAT_8X88680 | 8 | 129191272-129192787 | 252 | 83 | 9.31 | 8.01 | 0.1482 |
| *GASA18_qRT* | LSAT_9X31921 | 9 | 34435915-34437062 | 351 | 116 | 12.64 | 9.28 | -0.1767 |
| *GASA19_qRT* | LSAT_9X112021 | 9 | 182135163-182136755 | 276 | 91 | 10.28 | 9.27 | -0.2330 |
| *GASA20_qRT* | LSAT_0X26241 | U | 039433.1:357161-360126 | 1989 | 662 | 67.73 | 8.4 | -0.3287 |

z: molecular weight, y: Isoelectric point and x: grand average of hydropathicity
