# Supplementary material for: Genome-wide identification and characterization of the lettuce GASA family in response to abiotic stresses
Source: BMC Plant Biol. 2023 Feb 22;23:106. doi: 10.1186/s12870-023-04101-5 (PMC9945619; doi:10.1186/s12870-023-04101-5)
Supplement: Supplementary file 2 — Additional file 2: Fig. S1. A heat map of predicted subcellular localization of 20 LsGASA family. Nucl: nucleus, Cyto: cytoplasm, Mito: mitochondria, Cysk: cytoskeleton, Chlo: chloroplast, E.R: endoplasmic reticulum, Plas: plasma membrane, Golg: golgi apparatus, Pero: peroxisome, and Extra: extracellular. [file 12870_2023_4101_MOESM2_ESM.docx]

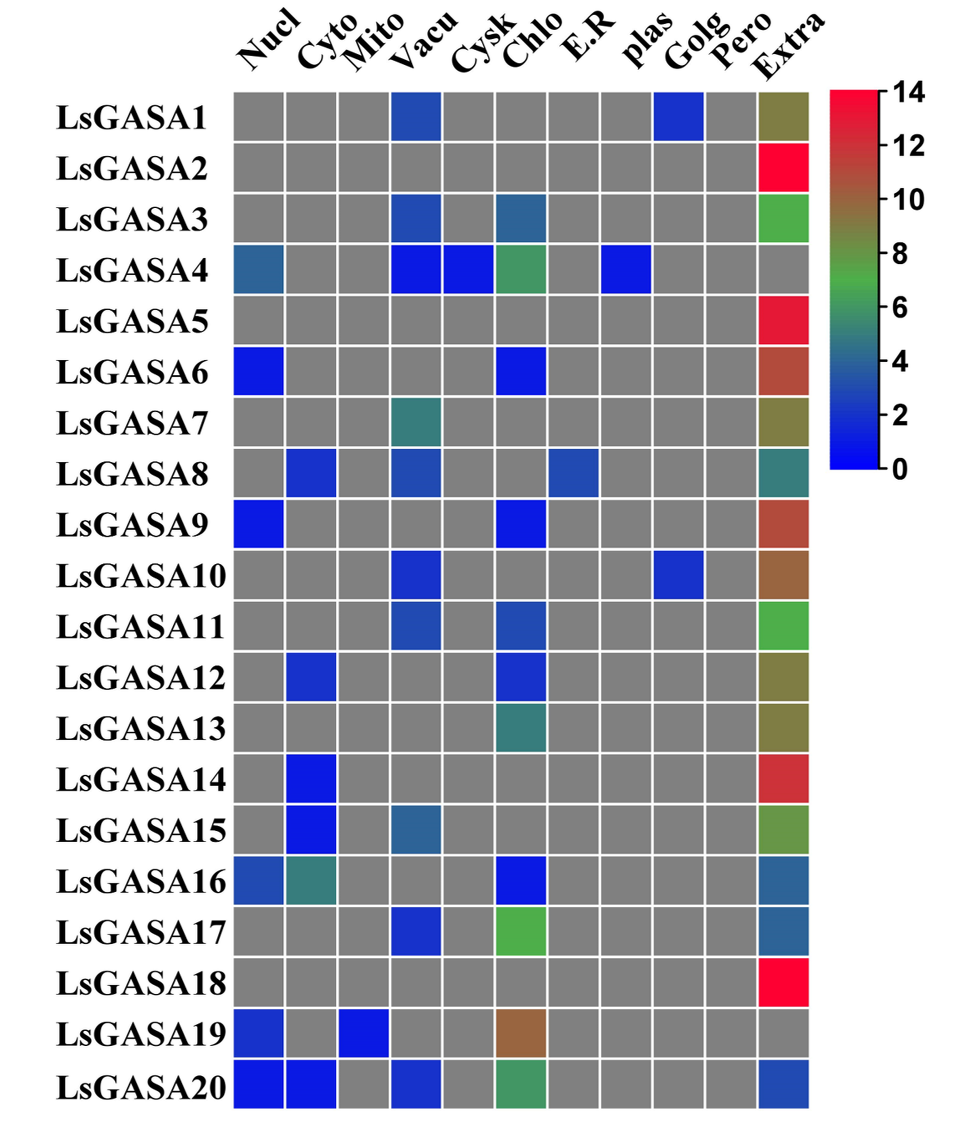


**Fig. S1.** A heat map of predicted subcellular localization of 20 *LsGASA* family. Nucl: nucleus, Cyto: cytoplasm, Mito: mitochondria, Cysk: cytoskeleton, Chlo: chloroplast, E.R: endoplasmic reticulum, Plas: plasma membrane, Golg: golgi apparatus, Pero: peroxisome, and Extra: extracellular.
