# Supplementary material for: Genome-wide identification and characterization of the lettuce GASA family in response to abiotic stresses
Source: BMC Plant Biol. 2023 Feb 22;23:106. doi: 10.1186/s12870-023-04101-5 (PMC9945619; doi:10.1186/s12870-023-04101-5)
Supplement: Supplementary file 3 — Additional file 3: Table S2. Ks, Ka, and Ka/Ks calculation and divergent time of the duplicated LsGASA pairs. [file 12870_2023_4101_MOESM3_ESM.docx]

**Table S2.** Ks, Ka, and Ka/Ks calculation and divergent time of the duplicated LsGASA pairs

| **Gene name** | **Ka^z^** | **Ks^y^** | **Ka/Ks ratio** | **MYA** | **Gene duplication** |
| --- | --- | --- | --- | --- | --- |
| *LsGASA9-LsGASA10* | 0.018221595 | 0.07073126 | 0.257617283 | 5.391102134 | Segmental |
| *LsGASA1-LsGASA11* | 0.055743795 | 0.2234 | 0.249524597 | 17.02743902 | Segmental |
| *LsGASA14-LsGASA19* | 0.2222 | 0.5154 | 0.431121459 | 39.28353659 | Segmental |
| *LsGASA15-LsGASA16* | 0.022799265 | 0.08083681 | 0.282040632 | 6.161342226 | Tandem |
| *LsGASA17-LsGASA18* | 0.2256 | 0.4517 | 0.499446535 | 34.42835366 | Segmental |
| *LsGASA3-LsGASA4* | 0.029409275 | 0.1262 | 0.233037044 | 9.618902439 | Tandem |
| *LsGASA6-LsGASA8* | 0.0338915 | 0.3333 | 0.101684668 | 25.40396341 | Segmental |
| *LsGASA12-LsGASA13* | 0.1302 | 0.2406 | 0.541147132 | 18.33841463 | Tandem |

z: synonymous substitution rate and y: non-synonymous substitution rate
