# Supplementary material for: Genome-wide identification and characterization of the lettuce GASA family in response to abiotic stresses
Source: BMC Plant Biol. 2023 Feb 22;23:106. doi: 10.1186/s12870-023-04101-5 (PMC9945619; doi:10.1186/s12870-023-04101-5)
Supplement: Supplementary file 4 — Additional file 4: Fig. S2. Semi qRT-PCR analysis of 20 LsGASA in shoot apical meristem (SAM) under heat stress conditions using ImageJ program. [file 12870_2023_4101_MOESM4_ESM.docx]

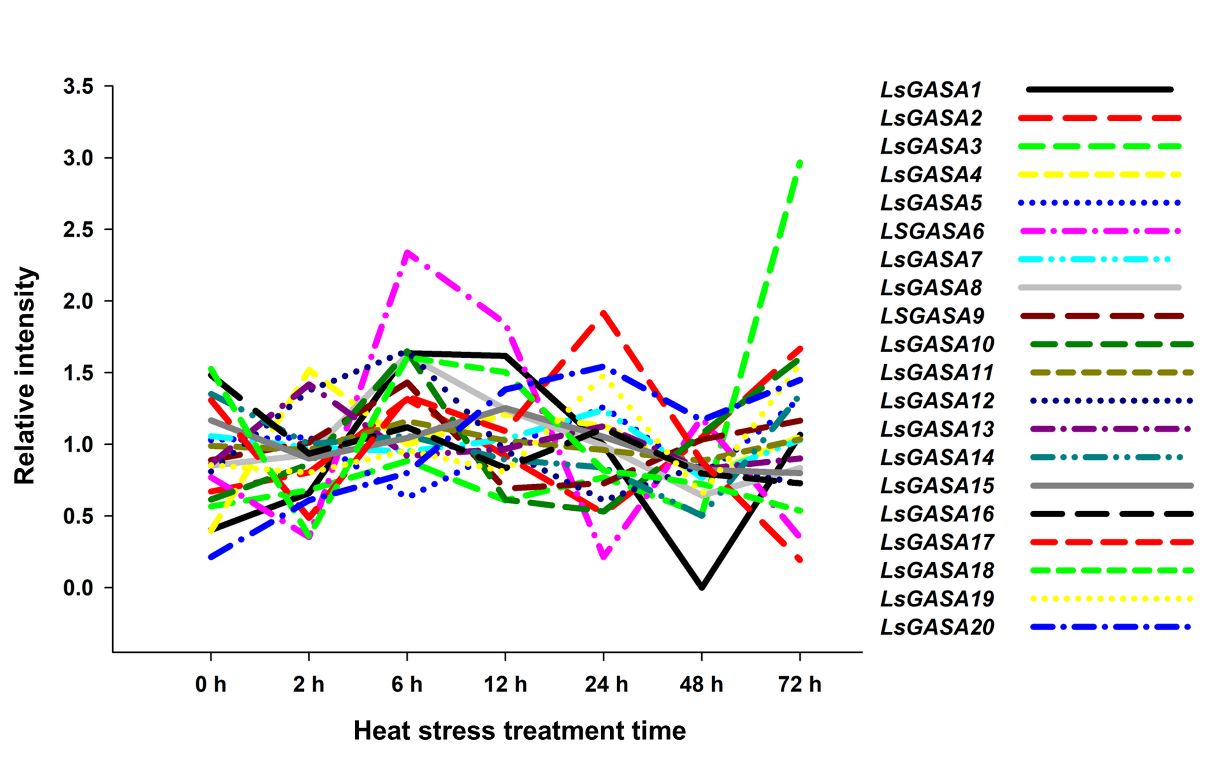


**Fig. S2.** Semi qRT-PCR analysis of 20 *LsGASA* in shoot apical meristem (SAM) under heat stress conditions using ImageJ program.
