# Supplementary material for: Genome-wide identification and characterization of the lettuce GASA family in response to abiotic stresses
Source: BMC Plant Biol. 2023 Feb 22;23:106. doi: 10.1186/s12870-023-04101-5 (PMC9945619; doi:10.1186/s12870-023-04101-5)
Supplement: Supplementary file 5 — Additional file 5: Fig. S3. Expression analysis of six LsGASA in different tissues (leaf, root, seed, stem and flower). Error bars represent the standard error of the mean. [file 12870_2023_4101_MOESM5_ESM.docx]

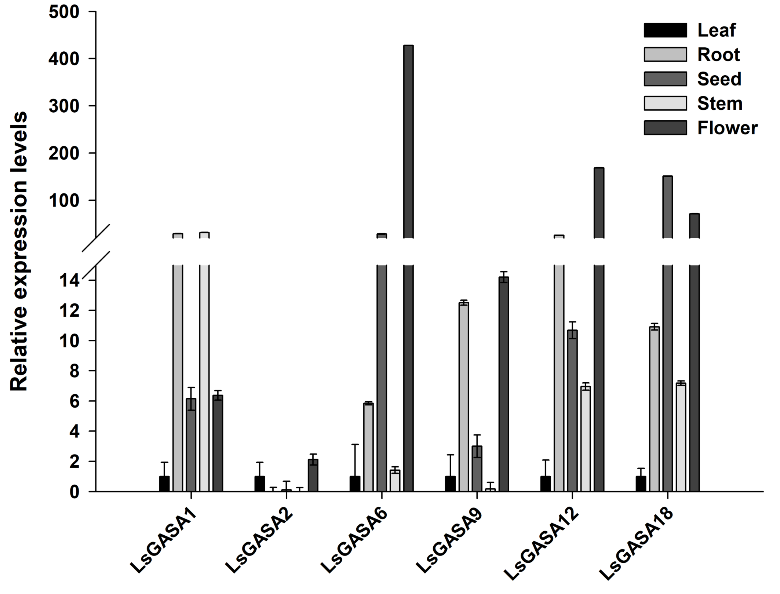


**Fig. S3.** Expression analysis of six *LsGASA* in different tissues (leaf, root, seed, stem and flower). Error bars represent the standard error of the mean.
