# Supplementary material for: Genome-wide identification and characterization of the lettuce GASA family in response to abiotic stresses
Source: BMC Plant Biol. 2023 Feb 22;23:106. doi: 10.1186/s12870-023-04101-5 (PMC9945619; doi:10.1186/s12870-023-04101-5)
Supplement: Supplementary file 6 — Additional file 6: Table S3. List of specific primers used in the study. [file 12870_2023_4101_MOESM6_ESM.docx]

**Table S3.** List of specific primers used in the study.

| Purpose | Gene name | Forward primer (5'→3') | Reverse primer (5'→3') | GeneID |
| --- | --- | --- | --- | --- |
| Gene expression | *UBQ21_qRT* | CAGGCATCAAGAGCAAGACT | TGGTCCCTTGATAAGTGCAG | LSAT_6X66261 |
|  | *GASA1_qRT* | GATGCTCGAAAGCAGGGTTA | TCATGTCCCGTGATAAGGGT | LSAT_1X23380 |
|  | *GASA2_qRT* | CATGTTGTGCTCGTTGTAGC | GGACACTTTCTTTTGCCACC | LSAT_2X9381 |
|  | *GASA3_qRT* | GCGAAATGCTTGTGTGTACC | TTGTCAATGAAACCCCTCCG | LSAT_2X69401 |
|  | *GASA4_qRT* | CAGGATCGTGCGTTGTATGA | GTCGGCGAGAAACTAACCTT | LSAT_2X69421 |
|  | *GASA5_qRT* | TATTGTGGGATCTGTTGCGG | ACTTGGATTTGCCCTTGGAG | LSAT_2X84540 |
|  | *GASA6_qRT* | TGGAAAACCAAGGAAGGTGG | CGTGAAATGGACCTCACAGT | LSAT_2X90361 |
|  | *GASA7_qRT* | GGAAGCATTCAAGGCCAAAC | CACACTGTTCTCTGTTGCCA | LSAT_3X49620 |
|  | *GASA8_qRT* | GACACAGTACCACAAGCCAT | TGTTGTAGCAAGGGCAAACT | LSAT_3X101621 |
|  | *GASA9_qRT* | TCTATGCAACAGGTCATGCG | TTGCATAGCAAGCACACGAT | LSAT_4X74100 |
|  | *GASA10_qRT* | GCAGCAAGGTGCAAACTATC | GCCTCGGGTGGTCATATTAG | LSAT_4X74180 |
|  | *GASA11_qRT* | ACTATGGCGGGTTCAAGTTT | TTTTTCGCCCTGCCTTTGAA | LSAT_4X129301 |
|  | *GASA12_qRT* | AGGTGGATCATTTGGATGCG | AACAGATCCCACACAGCTTC | LSAT_4X162200 |
|  | *GASA13_qRT* | TGGATTATTGCGGGATCTGC | TTGTTCCCTTGGGGTTCTTC | LSAT_4X162220 |
|  | *GASA14_qRT* | CTTGTGGAGTTCGTTGCTCT | GGTTTGCCATCGTGTGTTTT | LSAT_8X55660 |
|  | *GASA15_qRT* | TCTGCAACACACCATCGTAG | TTGTTTCCAATGGTACCGGA | LSAT_8X88640 |
|  | *GASA16_qRT* | GAAATCTCGATCGTCGGAGG | CTACGATGGTGTGTTGCAGA | LSAT_8X88660 |
|  | *GASA17_qRT* | TCTACTGGTTCATGGTGGTCA | TGACTACGATGGTGTGTTGC | LSAT_8X88680 |
|  | *GASA18_qRT* | CACCAAGATGCACGAGTAGG | GGAGGTACGCATAAGCATGT | LSAT_9X31921 |
|  | *GASA19_qRT* | CCTCTATGGGAAATCCAGCG | TCTCGCAGTACTTCATGCAC | LSAT_9X112021 |
|  | *GASA20_qRT* | AGCAACACCACCTGCTAATC | CTTGGAGCAATACTAGGCGG | LSAT_0X26241 |
| Full-length | *GASA1_qRT* | GATGCTCGAAAGCAGGGTTA | TCATGTCCCGTGATAAGGGT | LSAT_1X23380 |
|  | *GASA2_qRT* | CATGTTGTGCTCGTTGTAGC | GGACACTTTCTTTTGCCACC | LSAT_2X9381 |
|  | *GASA6_qRT* | TGGAAAACCAAGGAAGGTGG | CGTGAAATGGACCTCACAGT | LSAT_2X90361 |
|  | *GASA9_qRT* | TCTATGCAACAGGTCATGCG | TTGCATAGCAAGCACACGAT | LSAT_4X74100 |
|  | *GASA12_qRT* | AGGTGGATCATTTGGATGCG | AACAGATCCCACACAGCTTC | LSAT_4X162200 |
|  | *GASA18_qRT* | CACCAAGATGCACGAGTAGG | GGAGGTACGCATAAGCATGT | LSAT_9X31921 |
| Subcellular  localization | *GASA1_qRT* | GATGCTCGAAAGCAGGGTTA | TCATGTCCCGTGATAAGGGT | LSAT_1X23380 |
|  | *GASA2_qRT* | CATGTTGTGCTCGTTGTAGC | GGACACTTTCTTTTGCCACC | LSAT_2X9381 |
|  | *GASA6_qRT* | TGGAAAACCAAGGAAGGTGG | CGTGAAATGGACCTCACAGT | LSAT_2X90361 |
|  | *GASA9_qRT* | TCTATGCAACAGGTCATGCG | TTGCATAGCAAGCACACGAT | LSAT_4X74100 |
|  | *GASA12_qRT* | AGGTGGATCATTTGGATGCG | AACAGATCCCACACAGCTTC | LSAT_4X162200 |
|  | *GASA18_qRT* | CACCAAGATGCACGAGTAGG | GGAGGTACGCATAAGCATGT | LSAT_9X31921 |
